# 1-Methyl-4,5-dinitro-1*H*-imidazole

**DOI:** 10.1107/S1600536809047126

**Published:** 2009-11-14

**Authors:** Yong-Xiang Li, Xiao-Jun Wang, Jian-Long Wang

**Affiliations:** a School of Chemical Engineering and Environment, North University of China, Taiyuan, People’s Republic of China

## Abstract

In the title compound, C_4_H_4_N_4_O_4_, the two nitro groups are twisted with respect to the imidazole plane, making dihedral angles of 24.2 (3) and 33.4 (4)°. In the crystal structure, the mol­ecules are linked through non-classical inter­molecular C—H⋯O hydrogen bonds.

## Related literature

For the synthesis, see: Damavarapu *et al.* (2007 ▶). For the biological activity of polynitro­imidazole systems, see: Hofmann (1953 ▶); Breccia *et al.* (1982 ▶); Boyer (1986 ▶). For their detonation performance, see: Storm *et al.* (1990 ▶); Katritzky *et al.* (1993 ▶); Bulusu *et al.* (1995 ▶).
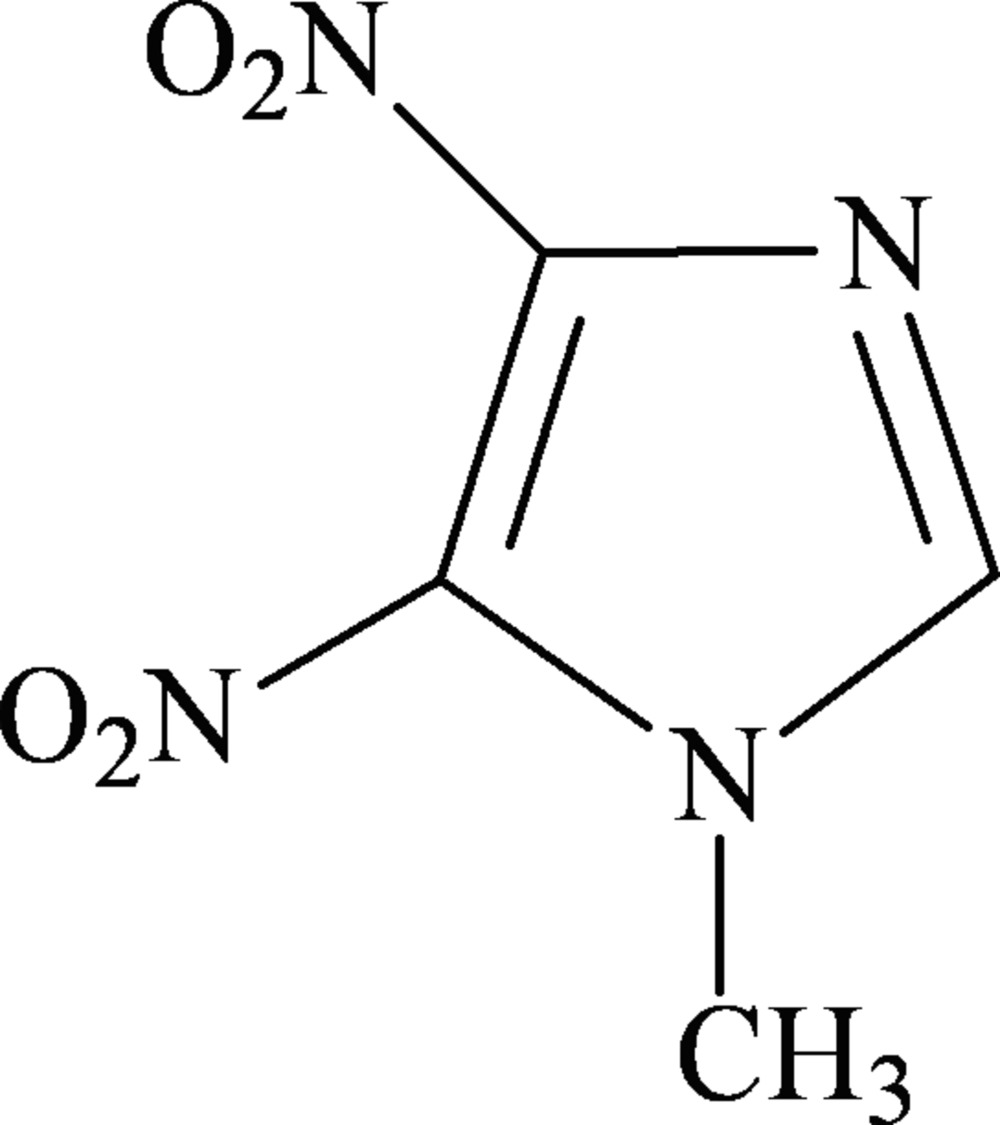


## Experimental

### 

#### Crystal data

C_4_H_4_N_4_O_4_
*M* *_r_* = 172.11
Orthorhombic, 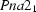

*a* = 8.412 (2) Å
*b* = 12.646 (3) Å
*c* = 6.563 (1) Å
*V* = 698.2 (3) Å^3^
*Z* = 4
Mo *K*α radiation
μ = 0.15 mm^−1^
*T* = 293 K
0.40 × 0.30 × 0.20 mm

#### Data collection

Rigaku R-AXIS RAPID IP diffractometer
Absorption correction: multi-scan (*ABSCOR*; Higashi, 1995 ▶) *T*_min_ = 0.944, *T*_max_ = 0.971
3573 measured reflections
871 independent reflections
648 reflections with *I* > 2σ(*I*)
*R*_int_ = 0.097

#### Refinement

*R*[*F* ^2^ > 2σ(*F* ^2^)] = 0.046
*wR*(*F* ^2^) = 0.112
*S* = 0.95
871 reflections
111 parameters
1 restraint
H-atom parameters constrained
Δρ_max_ = 0.22 e Å^−3^
Δρ_min_ = −0.18 e Å^−3^

### 

Data collection: *RAPID-AUTO* (Rigaku, 2000 ▶); cell refinement: *RAPID-AUTO*; data reduction: *CrystalStructure* (Rigaku, 2000 ▶); program(s) used to solve structure: *SHELXS97* (Sheldrick, 2008 ▶); program(s) used to refine structure: *SHELXL97* (Sheldrick, 2008 ▶); molecular graphics: *SHELXTL* (Sheldrick, 2008 ▶) and *DIAMOND* (Brandenburg, 1998 ▶); software used to prepare material for publication: *SHELXL97*.

## Supplementary Material

Crystal structure: contains datablocks I, global. DOI: 10.1107/S1600536809047126/lx2116sup1.cif

Structure factors: contains datablocks I. DOI: 10.1107/S1600536809047126/lx2116Isup2.hkl

Additional supplementary materials:  crystallographic information; 3D view; checkCIF report

**Fig. 1.**
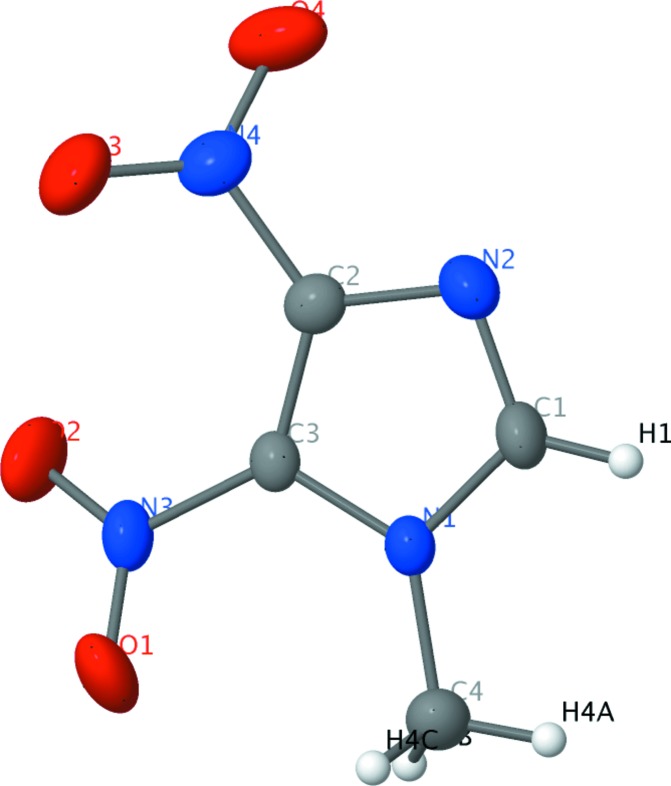
The molecular structure of the title compound with the atom numbering scheme. Displacement ellipsoids are drawn at the 30% probability level. H atoms are presented as a small spheres of arbitrary radius.

Enhanced figure: interactive version of Fig. 1

## Figures and Tables

**Table 1:** Hydrogen-bond geometry (Å, °)

*D*—H⋯*A*	*D*—H	H⋯*A*	*D*⋯*A*	*D*—H⋯*A*
C1—H1⋯O1^i^	0.93	2.49	3.150 (4)	128
C4—H4*A*⋯O4^ii^	0.96	2.48	3.428 (5)	170

Symmetry codes: (i) 
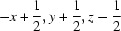
; (ii) 
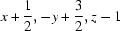
.

**Fig. 2.**
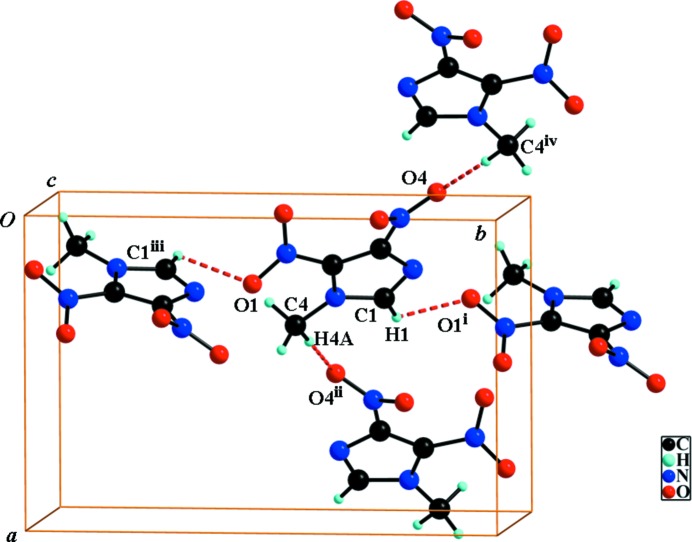
C–H···O interactions (dotted lines) in the crystal structure of the title compound. [Symmetry codes: (i) - x + 1/2, y + 1/2, z - 1/2; (ii) x + 1/2, - y + 3/2, z - 1; (iii) - x + 1/2, y - 1/2, z + 1/2; (iv) x - 1/2, - y + 3/2, z + 1.]

## supplementary crystallographic information

### Comment

Polynitroimidazole systems have been investigated extensively owing to their biological activity (Hofmann, 1953; Breccia *et al.*, 1982; Boyer, 1986). Recently, these so called "high energy density materials" have attracted renewed attention in conjunction with their favorable detonation performance (Storm *et al.*, 1990; Katritzky *et al.*, 1993; Bulusu *et al.*, 1995). As a promising candidate, 1-methyl-4,5- dinitroimidazole was synthesized by the nitration of *N*-methyl- imidazole (Damavarapu *et al.*, 2007). Here we reprot the crystal structure of the title compound (Fig. 1).

In the crystal structure, the two nitro groups are twisted with respect to the imidazole plane, making dihedral angles of 24.2 (3)° (N3/O1, O2) and 33.4 (4)° (N4/O3, O4). The molecular packing (Fig. 2) is stabilized by non-classical intermolecular C–H···O hydrogen bonds; the first between the imidazole H atom and an oxygen of the nitro group, with C1–H1···O^i^, the second between the methyl H atom and an oxygen of the nitro group, with C4–H4A···O4^ii^, respectively (Table 1).

### Experimental

The title compound was prepared according to literature method (Damavarapu *et al.*, 2007). Single crystals suitable for X-ray diffraction were obtained by evaporation of a solution of the title compound in methanol at room temperature.

### Refinement

All the Friedel pairs were merged. All H atoms were positioned geometrically and treated as riding, with C–H bond lengths constrained to 0.93 ° for imidazole ring H and 0.96 ° for methyl H atoms, and with *U*_iso_(H) = 1.2*U*_eq_(C) for imidazole ring H atom and 1.5*U*_eq_(C) for methyl H atoms.

### Crystal data

**Table:**

C_4_H_4_N_4_O_4_	*F*(000) = 352
*M**_r_* = 172.11	*D*_x_ = 1.637 Mg m^−^^3^
Orthorhombic, *P**n**a*2_1_	Mo *K*α radiation, λ = 0.71073 Å
Hall symbol: P 2c -2n	Cell parameters from 3573 reflections
*a* = 8.412 (2) Å	θ = 2.9–27.6°
*b* = 12.646 (3) Å	µ = 0.15 mm^−^^1^
*c* = 6.563 (1) Å	*T* = 293 K
*V* = 698.2 (3) Å^3^	Block, colorless
*Z* = 4	0.40 × 0.30 × 0.20 mm

### Data collection

**Table:**

Rigaku R-AXIS RAPID IP diffractometer	871 independent reflections
Radiation source: fine-focus sealed tube	648 reflections with *I* > 2σ(*I*)
graphite	*R*_int_ = 0.097
Detector resolution: 10.00 pixels mm^-1^	θ_max_ = 27.6°, θ_min_ = 2.9°
ω scans	*h* = −10→10
Absorption correction: multi-scan (*ABSCOR*; Higashi, 1995)	*k* = −16→16
*T*_min_ = 0.944, *T*_max_ = 0.971	*l* = −8→8
3573 measured reflections	

### Refinement

**Table:**

Refinement on *F*^2^	Secondary atom site location: difference Fourier map
Least-squares matrix: full	Hydrogen site location: difference Fourier map
*R*[*F*^2^ > 2σ(*F*^2^)] = 0.046	H-atom parameters constrained
*wR*(*F*^2^) = 0.112	*w* = 1/[σ^2^(*F*_o_^2^) + (0.075*P*)^2^] where *P* = (*F*_o_^2^ + 2*F*_c_^2^)/3
*S* = 0.95	(Δ/σ)_max_ = 0.001
871 reflections	Δρ_max_ = 0.22 e Å^−^^3^
111 parameters	Δρ_min_ = −0.18 e Å^−^^3^
1 restraint	Extinction correction: *SHELXL97* (Sheldrick, 2008), Fc^*^=kFc[1+0.001xFc^2^λ^3^/sin(2θ)]^-1/4^
Primary atom site location: structure-invariant direct methods	Extinction coefficient: 0.147 (18)

### Special details

**Table:**

Geometry. All esds (except the esd in the dihedral angle between two l.s. planes) are estimated using the full covariance matrix. The cell esds are taken into account individually in the estimation of esds in distances, angles and torsion angles; correlations between esds in cell parameters are only used when they are defined by crystal symmetry. An approximate (isotropic) treatment of cell esds is used for estimating esds involving l.s. planes.
Refinement. Refinement of F^2^ against ALL reflections. The weighted R-factor wR and goodness of fit S are based on F^2^, conventional R-factors R are based on F, with F set to zero for negative F^2^. The threshold expression of F^2^ > 2sigma(F^2^) is used only for calculating R-factors(gt) etc. and is not relevant to the choice of reflections for refinement. R-factors based on F^2^ are statistically about twice as large as those based on F, and R- factors based on ALL data will be even larger.

### Fractional atomic coordinates and isotropic or equivalent isotropic displacement parameters (Å^2^)

**Table:**

	*x*	*y*	*z*	*U*_iso_*/*U*_eq_	
C1	0.2730 (4)	0.7411 (2)	0.2853 (6)	0.0530 (8)	
H1	0.3251	0.7751	0.1789	0.064*	
C2	0.1370 (3)	0.7145 (2)	0.5486 (5)	0.0449 (7)	
C3	0.1843 (3)	0.61741 (19)	0.4808 (5)	0.0378 (6)	
C4	0.3530 (4)	0.5610 (3)	0.1731 (6)	0.0607 (9)	
H4A	0.3955	0.5988	0.0585	0.091*	
H4B	0.2780	0.5092	0.1262	0.091*	
H4C	0.4377	0.5264	0.2447	0.091*	
N1	0.2728 (2)	0.63562 (17)	0.3106 (4)	0.0410 (6)	
N2	0.1915 (4)	0.79129 (19)	0.4271 (5)	0.0561 (7)	
N3	0.1551 (3)	0.51403 (18)	0.5576 (4)	0.0490 (7)	
N4	0.0529 (3)	0.7419 (2)	0.7316 (5)	0.0569 (7)	
O1	0.2496 (3)	0.44510 (19)	0.5115 (6)	0.0806 (9)	
O2	0.0370 (3)	0.5008 (2)	0.6601 (5)	0.0773 (9)	
O3	0.0716 (4)	0.6874 (3)	0.8832 (5)	0.0878 (10)	
O4	−0.0300 (3)	0.8211 (2)	0.7273 (6)	0.0839 (10)	

### Atomic displacement parameters (Å^2^)

**Table:**

	*U*^11^	*U*^22^	*U*^33^	*U*^12^	*U*^13^	*U*^23^
C1	0.0718 (17)	0.0401 (15)	0.0470 (18)	−0.0025 (13)	0.0072 (16)	0.0127 (15)
C2	0.0424 (13)	0.0501 (16)	0.0421 (16)	0.0026 (10)	−0.0036 (12)	−0.0010 (13)
C3	0.0393 (12)	0.0394 (13)	0.0346 (13)	−0.0021 (9)	−0.0019 (11)	0.0059 (11)
C4	0.0676 (18)	0.0527 (19)	0.062 (2)	0.0108 (14)	0.0197 (17)	0.0053 (17)
N1	0.0471 (10)	0.0376 (12)	0.0384 (12)	0.0002 (9)	0.0032 (11)	0.0074 (11)
N2	0.0737 (16)	0.0423 (14)	0.0524 (18)	0.0039 (11)	0.0002 (15)	0.0042 (12)
N3	0.0560 (13)	0.0463 (14)	0.0446 (15)	−0.0099 (11)	−0.0002 (13)	0.0116 (12)
N4	0.0537 (13)	0.0690 (17)	0.0479 (16)	−0.0004 (14)	−0.0017 (13)	−0.0132 (15)
O1	0.0872 (16)	0.0502 (13)	0.104 (3)	0.0139 (11)	0.0169 (18)	0.0335 (15)
O2	0.0832 (18)	0.0780 (17)	0.071 (2)	−0.0301 (13)	0.0296 (16)	0.0049 (14)
O3	0.116 (2)	0.098 (2)	0.0499 (17)	0.0005 (17)	0.0167 (17)	0.0044 (15)
O4	0.0717 (14)	0.106 (2)	0.074 (2)	0.0307 (13)	−0.0068 (15)	−0.0273 (19)

### Geometric parameters (Å, °)

**Table:**

C1—N2	1.318 (5)	C4—N1	1.470 (4)
C1—N1	1.344 (4)	C4—H4A	0.9600
C1—H1	0.9300	C4—H4B	0.9600
C2—N2	1.337 (4)	C4—H4C	0.9600
C2—C3	1.365 (4)	N3—O2	1.212 (3)
C2—N4	1.436 (4)	N3—O1	1.218 (4)
C3—N1	1.361 (4)	N4—O3	1.220 (5)
C3—N3	1.423 (3)	N4—O4	1.222 (4)
			
N2—C1—N1	112.9 (3)	H4A—C4—H4C	109.5
N2—C1—H1	123.5	H4B—C4—H4C	109.5
N1—C1—H1	123.5	C1—N1—C3	105.7 (2)
N2—C2—C3	111.0 (3)	C1—N1—C4	124.1 (3)
N2—C2—N4	119.5 (3)	C3—N1—C4	130.2 (2)
C3—C2—N4	129.2 (3)	C1—N2—C2	104.5 (2)
N1—C3—C2	105.9 (2)	O2—N3—O1	125.1 (3)
N1—C3—N3	122.7 (2)	O2—N3—C3	117.8 (3)
C2—C3—N3	131.3 (3)	O1—N3—C3	117.2 (3)
N1—C4—H4A	109.5	O3—N4—O4	123.8 (4)
N1—C4—H4B	109.5	O3—N4—C2	118.8 (3)
H4A—C4—H4B	109.5	O4—N4—C2	117.3 (3)
N1—C4—H4C	109.5		
			
N2—C2—C3—N1	0.4 (3)	C3—C2—N2—C1	−0.3 (4)
N4—C2—C3—N1	−174.1 (3)	N4—C2—N2—C1	174.8 (3)
N2—C2—C3—N3	−179.5 (3)	N1—C3—N3—O2	−155.1 (3)
N4—C2—C3—N3	6.0 (5)	C2—C3—N3—O2	24.8 (5)
N2—C1—N1—C3	0.2 (4)	N1—C3—N3—O1	23.5 (4)
N2—C1—N1—C4	179.3 (3)	C2—C3—N3—O1	−156.6 (3)
C2—C3—N1—C1	−0.3 (3)	N2—C2—N4—O3	−143.7 (4)
N3—C3—N1—C1	179.6 (3)	C3—C2—N4—O3	30.5 (5)
C2—C3—N1—C4	−179.4 (3)	N2—C2—N4—O4	34.1 (4)
N3—C3—N1—C4	0.5 (5)	C3—C2—N4—O4	−151.8 (3)
N1—C1—N2—C2	0.1 (4)		

### Hydrogen-bond geometry (Å, °)

**Table:**

*D*—H···*A*	*D*—H	H···*A*	*D*···*A*	*D*—H···*A*
C1—H1···O1^i^	0.93	2.49	3.150 (4)	128
C4—H4A···O4^ii^	0.96	2.48	3.428 (5)	170

Symmetry codes: (i) −*x*+1/2, *y*+1/2, *z*−1/2; (ii) *x*+1/2, −*y*+3/2, *z*−1.
